# Annoying Psoriasis and Atopic Dermatitis: A Narrative Review

**DOI:** 10.3390/ijms23094898

**Published:** 2022-04-28

**Authors:** Wei-Yu Chen, Shao-Chuan Chen, Shou-Yi Hsu, Yu-An Lin, Chun-Ming Shih, Chun-Yao Huang, Kuo-Hsien Wang, Ai-Wei Lee

**Affiliations:** 1 Department of Anatomy and Cell Biology, School of Medicine, College of Medicine, Taipei Medical University, Taipei 11031, Taiwan; b101102094@tmu.edu.tw (W.-Y.C.); b101107027@tmu.edu.tw (S.-C.C.); b101109018@tmu.edu.tw (S.-Y.H.); b101109022@tmu.edu.tw (Y.-A.L.); 2 Department of Family Medicine, Shuang Ho Hospital, Taipei Medical University, New Taipei 11031, Taiwan; 3 Department of Internal Medicine, School of Medicine, College of Medicine, Taipei Medical University, Taipei 11031, Taiwan; cmshih53@tmu.edu.tw (C.-M.S.); cyhuang@tmu.edu.tw (C.-Y.H.); 4 Cardiovascular Research Center, Taipei Medical University Hospital, Taipei 11031, Taiwan; 5 Taipei Heart Institute, Taipei Medical University, Taipei 11031, Taiwan; 6 Department of Dermatology, Taipei Medical University Hospital, Taipei 11031, Taiwan; khwang40@gmail.com

**Keywords:** psoriasis, atopic dermatitis

## Abstract

Skin is an important organ that mainly functions as a barrier. Skin diseases can damage a person’s self-confidence and reduce their willingness to socialize, as well as their social behavior and willingness. When the skin appearance is abnormal, in addition to affecting the quality of life, it often leads to personal, social, and psychological dysfunction and even induces depression. Psoriasis and atopic dermatitis are common chronic skin diseases. Their prevalence in the world is 3–10%, and there is an increasing trend year by year. These congenital or acquired factors cause the dysfunction of the immune system and then destroy the barrier function of the skin. Because these patients are flooded with a variety of inflammatory mediators, this causes skin cells to be in chronic inflammation. Therefore, psoriasis and atopic dermatitis are also considered systemic chronic inflammatory diseases. In the healthcare systems of developed countries, it is unavoidable to spend high costs to relieve symptoms of psoriasis and atopic dermatitis patients, because psoriasis and atopic dermatitis have a great influence on individuals and society. Giving a lot of attention and developing effective treatment methods are the topics that the medical community must work on together. Therefore, we used a narrative review manuscript to discuss pathogenesis, clinical classification, incidence, and treatment options, including topical medication, systemic therapeutics, immunosuppressive medication for psoriasis, and atopic dermatitis, as well as also comparing the differences between these two diseases. We look forward to providing readers with comprehensive information on psoriasis and atopic dermatitis through this review article.

Psoriasis and atopic dermatitis are common, chronic inflammatory skin diseases that are clinically different. Their prevalence in the world is 3–10%, and there is an increasing trend year by year. However, in the young population, PS is commonly mistaken for AD [1]. Although the two are different diseases, they share some common features, such as infiltration of immune cells in the skin, altered expression of some proinflammatory cytokines, genetic factors, environmental influences, and barrier alterations [2]. Psoriasis shows eczematous in the acute phase, while AD can display psoriasiform in the resistant stage. So, it is very important to discuss these two diseases.

## 1. Morbidity and Clinical Classification

### 1.1. Morbidity and Clinical Classification of Psoriasis

Psoriasis is estimated to affect 1–3% of the population worldwide. The prevalence of psoriasis is highly variable across the globe, ranging from 0.05% to 11.90% depending on race and geographic location [3]. Throughout our lifetime, incidences of psoriasis have increased in a roughly linear pattern, from 0.12% in the first year to 1.2% after 18 years. A variety of environmental factors are thought to correlate with psoriasis prevalence, including climate, sun exposure, and humidity level. It has been found that psoriasis prevalence peaks in late winter, and dips in the summer. This may be attributed to reduced ultraviolet radiation (UVR) exposure, lowered temperature, darkness, and lowered humidity worsening the disease [4]. According to National Health and Nutrition Examination Survey data gathered from 2009 through 2010, the prevalence of psoriasis among those over the age of 20 was 3.1–3.2% (95% CI 2.6–3.7%) in the USA. There was no difference in prevalence between men and women, but the odds of psoriasis were significantly increased in the white population compared with those in the non-white population [5]. The prevalence of phenotypes, from high to low, are as follows: plaque, 55%; scalp, 52%; palmar, 14%; nail, 23%; inverse, 21% [6]. In South America, one randomized telephone survey of residents from all 26 Brazilian State capitals reported a psoriasis prevalence of 1.31% [7]. In Europe, the prevalence of psoriasis in adults varies from 2.80% to 11.90%, with the United Kingdom having the lowest prevalence (2.80%), and Norway having the highest prevalence (11.90%) [8]. Other countries had prevalence rates of 2.22–9.2% (Denmark), 1.80–3.10% (Italy), 5.20% (95% CI: 4.68–5.72, France), and 9.4% (Sweden) [9]. It is important to note that the lack of studies in developing countries may cause an underestimation of the prevalence of psoriasis and can limit our understanding of the situation in those areas. The prevalence of psoriasis reported in these studies can be variable, even within the same geographic location or in members of the same race, due to the case definition (self-report, physician’s diagnosis, and dermatologist’s diagnosis), the method of assessment (questionnaires, clinical examination, and registry data), and the definition of prevalence (point, period, and lifetime) [10].

Clinical presentation of psoriasis varies. There are five subtypes of psoriasis, including plaque psoriasis, guttate psoriasis, erythrodermic psoriasis, inverse psoriasis, and pustular psoriasis. Plaque psoriasis, also known as psoriasis vulgaris, is the most prevalent, with about 90% of psoriasis patients having this type. The clinical manifestations are well-demarcated, erythematous, and pruritic plaques, as well as xerotic skin covered in silvery scales. Lesions can be round, oval, or irregular, and smaller lesions may merge into a single lesion covering a large area. They are most commonly located on the scalp, trunk, lumbosacral area, and extensor surfaces of the limbs. Scraping of scales results in small bleeding points, known as the Auspitz sign. About 80% of patients have a milder disease presentation, which covers less than 10% of the body surface, while the other 20% suffer from more severe symptoms and may have extracutaneous manifestations [11]. The most common nondermatological manifestations of psoriasis are psoriatic arthritis and nail diseases. Psoriatic arthritis affects approximately 30% of cutaneous psoriasis patients, 12 years after disease onset (on average). It presents as inflammation at tendons or ligaments, often at insertion sites into bones, involving the fingers, wrists, toes, and ankles [12].

### 1.2. Morbidity and Clinical Classification of Atopic Dermatitis

#### 1.2.1. Morbidity of Atopic Dermatitis

Many studies have been performed to determine the prevalence of childhood AD worldwide. However, the different diagnostic criteria and methodologies used by different centers and countries make it difficult to compare the results of studies and identify the true prevalence of AD [13]. The International Study of Asthma and Allergies in Childhood (ISAAC) uses a standardized and validated methodology to provide a comprehensive understanding of allergy prevalence and severity worldwide [14]. This study revealed that over 20% of children are affected by AD in some countries, but there were significant differences in prevalence between different regions, countries, and even centers in the same city [15]. The ISAAC Phase III study analyzed the data of 385,853 children aged 6–7 years and 663,256 children aged 13–14 years. The results showed that the prevalence of AD ranged from 0.9% in India to 22.5% in Ecuador for the former age group and from 0.2% in China to 24.6% in Colombia for the latter age group [16]. Prevalence values were significantly higher in Scandinavia, Northern and Western Europe, Australasia, and urban areas in Africa and were lower in Eastern Europe, the Middle East, China, and Central Asia. In the USA, the overall prevalence of AD was 10.7%. According to a study conducted by the National Survey of Children’s Health, 9752/102,353 children at or below the age of 17 were diagnosed with AD in the United States [17]. The data reported a wide range of prevalence between 8.7% and 18.1%, with higher prevalence values in many of the East Coast states, which may be associated with urban living. According to the ISAAC Phase III study in the Asia-Pacific region, AD prevalence in children aged between 13 and 14 years ranged from 0.9% in China to 9% in Singapore and Malaysia. Recent studies have shown increasing AD prevalence in developing countries in this region. In China, one study based on clinical diagnoses by dermatologists, which included 13,998 preschool children from 12 large cities, showed that the prevalence of AD was 12.94% [18]. A cross-sectional survey conducted by the health centers of elementary schools in Taipei City demonstrated that the 12-month prevalence of AD was 10.7% among children aged 6–8 years in 2007–2008 (Ho C-L, et al., The prevalence and risk factors of atopic dermatitis in 6–8 year-old first graders in Taipei, Pediatrics and Neonatology 2018). While a greater understanding of childhood AD prevalence has been provided by ISAAC and many other studies, there are a limited number of studies on adult AD. Though AD has been thought to be more prevalent in children, a systematic review and meta-analysis of seven birth cohort studies showed no significant difference in AD prevalence between childhood and early adulthood. This finding may help explain how the disease persists from childhood, its remission process, and its adult-onset in some [19].

AD is more prevalent than psoriasis, and a higher prevalence of AD has been found in the younger population. In adults, these two diseases seem to have a similar prevalence. It can be difficult to differentiate AD from psoriasis, especially psoriasiform AD. Some studies even found that AD and psoriasis could co-exist, and the prevalence of concomitant AD and psoriasis was 1.3% of the total population of patients diagnosed with AD or psoriasis.

#### 1.2.2. Clinical Classification of Atopic Dermatitis

In general, atopic dermatitis is a chronic disease characterized by recurrent eczematous skin, with pruritus, excoriation, and dry skin. While AD can be divided into several subtypes according to the age of onset, different clinical appearances, and expression of different molecules, the typical clinical presentation of atopic dermatitis is well established [20].

A. Lichenoid atopic dermatitis
  In general, the clinical manifestations of Lichenoid atopic dermatitis include thickening, raised and uneven skin, because of continuous itch and long-term friction, it will show a leather-like appearance [21].
B. Juvenile plantar dermatosis
  Juvenile plantar dermatosis mainly occurs on the soles of children and adolescents. Different from athlete’s foot caused by a fungal infection, the typical expressions are extremely itchy, shiny appearance, and erythema on the surface of the first toe and sole [22]. Wearing plastic shoes easily exacerbates disease
C. Nummular-type atopic dermatitis
  Nummular-type atopic dermatitis is a chronic disease. Clinically, multiple coin-shaped lesions can be seen on the skin [22], these lesions are itchy and well-defined. This kind of dermatitis often occurs after skin injuries, such as burns, and contusions, most of which can be successfully treated with steroids.
D. Follicular atopic dermatitis
  The clinical presentations of follicular atopic dermatitis are mostly single papules, and the lesions often involve the hair follicles and surrounding dermis [20]. In children, the lesions often include the entire chest, back, abdomen, and proximal limbs. Doctors usually recommend topical steroids for 2 to 4 weeks [22].
E. Eczema coxsackium
  Eczema coxsackium is a specific term for Coxsackie virus infection. This type of dermatitis is caused by Coxsackie infection in children with AD [22].
  Pustules and oral ulcers occur in the anterior elbow and popliteal fossa [23]. Therefore, it is recommended to supplement the lost water and electrolytes in clinical practice, and treat the symptoms of the patient.
F. Psoriasiform atopic dermatitis
  Psoriasis and atopic dermatitis are the two different diagnoses, and psoriasiform atopic dermatitis, as the name suggests, is atopic dermatitis with a tissue type similar to psoriasis, which simultaneously manifests the characteristics of two diseases, such as itching and desquamation [24]. The use of traditional steroids was found to be ineffective and phototherapy was necessary to improve lesions [22].

## 2. Molecular Mechanisms

### 2.1. Pathological Molecular Mechanisms of Psoriasis

Psoriasis is a chronic inflammatory disease involving complex interactions between different types of cells, as well as numerous cytokines and chemokines, and is strongly associated with genetic predisposing factors [25]. The resulting state of chronic inflammation results in hyper-proliferation and dysfunctional differentiation of keratinocytes (KCs). Histologically, psoriatic lesions display neovascularization through infiltration by inflammatory cells, including dendritic cells (DCs), macrophages, and T cells [26]. Dysfunction of the innate and adaptive immune systems results in sustained inflammation. It has also been reported that the TNFα-IL23-Th17 axis plays a major role in chronic inflammation, leading to the development of psoriatic lesions [27]. In their initiation phase, psoriatic lesions can be triggered by infection, trauma, and medications. Injured KCs release self-DNA, self-RNA, and antimicrobial peptides, including LL-37, b-defensins, and S100 protein. LL-37 combines with self-DNA to form LL-37-DNA complexes, which are recognized by toll-like receptor 9 to activate plasmacytoid DCs (pDCs), which in turn produce type I interferons (IFNα and IFNβ) [28]. Type I IFNs from pDCs and injured cells subsequently stimulate myeloid DCs (mDCs) to mature and produce TNFα, IL23, and IL12, which further contribute to the activation of T helper type 1 and T helper type 17 cells (Th1 and Th17 cells). Th1 and Th17 cells produce multiple types of cytokines, including IFNγ and IL17A, respectively, which stimulate KC proliferation. The assorted cytokines and chemokines produced also lead to the aggregation and activation of inflammatory cells [29]. Additionally, LL-37 binds to self-RNA to form LL-37-RNA complexes, which activate mDC via toll-like receptor 7/8 and drive monocytes to secrete large amounts of TNFα, IL23, and IL12 [28]. IL23, composed of the subunits p19 and p40, plays an important role in psoriasis [30]. IL23 transmits signals through JAK2/TYK2 and STAT in innate and adaptive immune cells, thereby modulating the expression of pro-inflammatory cytokines, including IL17A. IL17A/F binds to receptors on KCs, resulting in the recruitment of ACT1 adaptor protein. This, together with the IL17 receptor complex, activates a series of kinases in the affected cell. These activated kinases signal through nuclear factor-kB (NF-kB), which drives the transcription of pro-inflammatory cytokines, chemokines, and antimicrobial peptides [25,26]. IL17A also enables KCs to produce more chemokines that drive the aggregation of neutrophils in the epidermis. IL36 belongs to the IL1 superfamily and includes three agonists: IL36α, IL36β, and IL36γ [31]. IL36γ expression is high in psoriatic lesions of psoriasis patients. IL36γ, together with IL17A, stimulates KCs to secrete TNFα. In mouse experiments, mice without IL36 receptors lack IL17-producing γδT cells in the body, which implies that IL36γ is inevitable in the TNFα-IL23-IL17 axis [31,32,33]. Single transducer and activator of transcription 3 (STAT3) play an important role in psoriasis. STAT3 regulates the development and polarization of T cells. Genes encoding for the antiviral response gene-regulated innate immune system have recently been found to be strongly associated with psoriatic susceptibility, and genes strongly associated with adaptive immunity are variations in genes encoding nuclear factor-kB (NF-kB), CARD, and type I IFNs [11]. These genes participate in the mechanisms of psoriasis by modulating the immune system. For example, they increased the expression of IL17, Th2-related genes, and immune checkpoint genes. Additionally, Notch signaling plays a major role in the regulation of embryonic development. Notch signaling is essential for keratinocyte differentiation. Notch1 is expressed in all epidermal layers, Notch2 in the basal cell layer, and Notch3 in basal cell and spinous cell layers in the normal epidermis [34]. In psoriasis patients, there are highly expressed Notch1 and Hes-1 mRNA levels [35]. Notch1 signaling may contribute to the pathogenesis of psoriasis by microRNA 125b [34]. Some studies have suggested that the microbiome may be a potential trigger in the pathophysiology of psoriasis. However, it is still unknown whether microbial changes on the skin of psoriasis patients have a causal association with, or are merely the consequence of, the inflammatory microenvironment [36]. Compared to that of normal skin in healthy individuals, microbiome diversity decreases on the skin of psoriasis patients. In lesioned skin, certain types of bacteria such as *Staphylococcus aureus* and *Streptococcus* increase, while levels of *Staphylococcus epidermitis*, *Propionibacterium*, *Malassezia*, and *Cutibacterium* decrease [36,37]. These microbiomes may result in T cells differentiating into Th17 cells, exacerbating skin inflammation through the Th17 axis [38]. However, the relationship between the pathogenesis of psoriasis and the microbiome warrants further research [11].

### 2.2. Pathological Molecular Mechanisms of Atopic Dermatitis

Atopic dermatitis (AD) is a common chronic skin disorder that causes pruritic and eczematous skin lesions and usually begins in childhood. The pathogenesis of AD, which is still under investigation, is multifactorial and involves complex interactions between genetic, environmental, and immunological factors. In a recent study, the main contributing factors driving disease development were identified as skin barrier dysfunction, immune dysregulation, and microbiome changes [39].

Among them, the theory of skin barrier dysfunction is the most important and is thought to be the major factor in the pathogenesis of AD. The skin of healthy individuals can serve as a physical antimicrobial barrier and prevent water loss. It also enriches nonpathogenic bacterial flora, primarily through the outermost layer, stratum corneum, and lamellar sheets, which provide them with a supporting matrix. Skin barrier impairment can result from both intrinsic and extrinsic causes. Genetics play an important role in the normal function of the skin barrier, and multiple genes have been found to be associated with AD. Loss-of-function mutations on the filaggrin (FLG) gene, which is located in the epidermal differentiation complex on chromosome 1q21, have been found to lead to a decrease in filaggrin products, which are present in KCs that cause keratin filaments to aggregate. FLG deficiency and FLG degradation products facilitate allergen sensitization and increase infection risk and transepidermal water loss. Studies have also shown that children have a higher risk of AD when their mothers have FLG mutations, indicating a maternal inheritance pattern [40]. There are other genes associated with AD as well, such as Filaggrin 2 (*FLG2*) and Serine Peptidase Inhibitor Kazal Type 5 (*SPINK5*). The latter of these encodes a protease inhibitor that contributes to epidermal structural integrity. Mutations in SPINK5 correlate with early-onset AD [41]. Immune dysregulation plays a crucial role in the pathogenesis of AD. Once the skin barrier is disrupted, the epidermis releases thymic stromal lymphopoietin, triggering Th2 and Th22 immune responses. In the acute phase of AD, lesional skin is characterized by infiltration of inflammatory cells, particularly Th2 and Th22 cells, increasing expression of cytokines and chemokines such as IL4, IL13, and IL22. Such cytokines and chemokines suppress the function and production of terminal proteins, such as FLG and loricrin [42]. These Th2 products also enhanced IgE production in B cells, which caused elevated serum IgE levels in over 80% of AD patients [43]. Moreover, high serum IgE levels were associated with IgE autoreactivity and increased AD severity. Chronic AD lesions are characterized by a switch of the immune response from Th2 to Th1, as well as activation of Th17 and Th22 cells, leading to remodeling and fibrosis of skin tissue [44]. Upregulation of terminal differentiation gene expression, particularly of S100A8 and S100A9, is also observed in AD lesional skin. S100A8 and S100A9, whose secretion by activated KCs is primarily induced by Th17 cytokines, can act as damage-associated molecular pattern molecules that increase IL33 production and amplify Th2 immune responses [45]. In addition, thymic stromal lymphopoietin and IL31 produced by Th2 cells induce pruritic sensation, with scratching potentially leading to further physical damage to the skin barrier, facilitating the penetration of irritants and allergens [46].

## 3. Management

### 3.1. Treatment Options for Psoriasis

There are a variety of options for the treatment of psoriasis. Treatment strategies are determined by the severity of the disease and the patient’s preference. Management of mild psoriasis starts with topical therapies, including corticosteroids, vitamin D analogs, and retinoids. Among them, corticosteroids can be used as monotherapy, while vitamin D analogs and retinoids are usually combined with steroids [47]. Phototherapy, including ultraviolet B, psoralen ultraviolet A, and pulsed dye laser, as well as photodynamic therapy and light-emitting diodes, are used for treating stable psoriatic lesions, refractory psoriasis plaques, and moderate-to-severe psoriasis patients [48]. Methotrexate, cyclosporine, and acitretin are the most frequently used systemic agents for patients with severe psoriasis [49]. Psoriasis is a T cell-mediated disease, with the TNFα-IL23-IL17 axis playing a primary role in inflammatory status, leading to psoriatic lesions on the skin. Thus, treatments using biologics to target these cytokines have been developed in recent decades. However, due to their lower cost, patients usually accept conventional therapies (methotrexate and phototherapy) before shifting to biologics [27].

#### 3.1.1. Interleukin 23-Targeted Therapies

A. Tildrakizumab is a humanized IgG1κ monoclonal antibody to the IL23 p19 subunit [50]. Two Phase III clinical trials (reSURFACE1 and reSURFACE2) revealed that tildrakizumab was more potent than etanercept and had a favorable safety profile [51].

B. Guselkumab is a monoclonal antibody targeting IL23 p19. According to two phase III trials (VOYAGE 1 and VOYAGE 2), guselkumab is an effective and safe novel biologic. Out of three cytokines with activity leading to psoriasis (IL23 p19, TNFα, and IL17), IL23 p19 may be the most specific, as fewer adverse events occurred in patients treated with guselkumab [52].

C. Ustekinumab targets the p40 subunit shared by IL12 and IL23. A phase III trial evaluating the efficacy and safety of ustekinumab elucidated that this drug had good effectiveness, as well as a good safety profile in a 5-year follow-up [53]. The trial also revealed that ustekinumab was a cost-effective treatment, due to few side effects and adverse events [54].

D. Risankizumab targets IL23 p19. Two phase III trials (UltIMMa-1 and UltIMMa-2) compared risankizumab with ustekinumab and a placebo in patients with moderate-to-severe psoriasis. The results included data suggesting that risankizumab was safer and more efficient than ustekinumab and the placebo [55].

#### 3.1.2. Interleukin 17-Targeted Therapies

A. Brodalumab is a fully human IgG2 monoclonal antibody that targets the IL17 receptor. This disrupts the IL17 pathway by blocking the activity of IL17A/E/F and shows high efficacy in the rapid improvement of psoriasis [56].

B. Xekizumab is a humanized monoclonal antibody that selectively binds to IL17A. A two-phase, prospective, double-blind, phase III randomized trial (UNCOVER-2 and UNCOVER-3) compared the efficacies of xekizumab, etanercept, and a placebo, revealing that the efficacy of xekizumab was significantly greater than that of etanercept and the placebo [57].

C. Secukinumab, which targets IL17A, is the fastest-acting treatment among these therapies, and it achieves the highest rate of PASI 100 response. In the DERMBIO registry, all moderate to severe psoriasis patients treated with biologics in Denmark since 2007. The data demonstrate that adverse events occurred most frequently in patients treated with secukinumab, which had the lowest drug survival rate of all the biologics included [58]. Because of its fast action, efficacy, and favorable safety profile, secukinumab is the first-line biologic for psoriasis [29].

D. Bimekizumab is a novel monoclonal antibody with bispecific neutralizing properties against both IL17A and IL17F [59]. It showed promising results for treating patients with mild psoriasis, with good patient responses and few adverse events [60]. In a randomized, double-blinded, placebo-controlled phase IIb trial, 250 patients with moderate to severe psoriasis either received bimekizumab every four weeks in doses of 64, 160 (both with and without a 320 mg loading dose), 320, or 480 mg, or received the placebo. The results demonstrated that the efficacy of each bimekizumab dose group was superior to that of corresponding doses of the placebo and that the highest efficacy was observed in the 320 mg dose group. Patients treated with bimekizumab also tolerated the treatment well, experiencing few side effects [59].

The above IL17-targeted agents were more effective when used frequently in high doses than when they were used infrequently in low doses. It is noteworthy that IL17 inhibitors were less tolerant than other biological agents. Although recent data showed a connection between IL17 signaling deficiencies and psychiatric disease, IL17-targeting therapies are efficient and are safe treatment choices.

#### 3.1.3. Tumor Necrosis Factor-Targeted Therapies

A. Adalimumab, which blocks the p55 and p75 cell surface TNF receptors, is a well-tolerated, effective, and generally safe treatment option for adults with moderate to severe psoriasis, as well as for children and adolescents [61].

B. Certolizumab, a monoclonal antibody targeting TNF, showed significant improvement in the treatment of moderate to severe chronic plaque psoriasis. The higher dose regimens were observed to have greater effects without resulting in a significant increase in adverse events [62].

C. Etanercept is another TNF-targeted psoriasis therapy. Using prospective data from two phase III trials, etanercept was found to be safe and effective for adults with psoriasis [63]. A similar finding was observed in the pediatric population. Greater clinical improvement was shown when etanercept was combined with other systemic therapies, including methotrexate, acitretin, ciclosporin, and narrowband UVB phototherapy than when it was used as monotherapy [64].

Recent data revealed promising results suggesting that IL17, IL12/23, and IL23 inhibitors are highly effective for short-term use. A meta-analysis of 28 studies showed that IL17 inhibitors had higher efficacy but relatively poor safety; in contrast, risankizumab had a lower risk of inducing adverse effects. Confirmation of the long-term clinical efficacy and safety of biologics requires further research. In a meta-analysis, including 24 randomized placebo-controlled trials, ustekinumab, secukinumab, ixekizumab, brodalumab, guselkumab, and tildrakizumab were determined to be clinically tolerable, safe, and efficient in treating moderate to severe plaque psoriasis [65]. Another systematic review, including 109 studies enrolling a total of 39,882 people, demonstrated that biologics, such ixekizumab, secukinumab, brodalumab, guselkumab, certolizumab, and ustekinumab were more potent than conventional drugs, such corticosteroids, vitamin D analogs, and retinoids [66]. So far, there are a variety of anti-psoriatic biologics that offer a greater benefit/risk ratio than conventional formulations for treating obstinate psoriasis (Table 1).

### 3.2. Treatment Options for Atopic Dermatitis

Basic skincare plays a primary role in the restoration and maintenance of skin barrier function in patients with AD. Appropriate bathing habits include using warm water, instead of hot water, and less irritating soap. The use of synthetic cleansers with a low pH can reduce skin irritation by preserving skin acidity. Topical emollients containing urea, glycerin, and ceramides have been found to be effective in decreasing transdermal water loss (TEWL), which leads to hydration of the stratum corneum and a reduction in the SCORAD index score of affected skin [75].

#### 3.2.1. Topical Medication

A. Topical Corticosteroids (TCSs) have been the mainstay treatment for AD. They suppress many inflammatory cells and cytokines, including neutrophils, monocytes, lymphocytes, LCs, IL2, TNF, and GM-CSF, resulting in anti-inflammatory and immunosuppressive effects. They are widely used and have been the recommended first-line therapy for acute exacerbation of AD for several decades. In general, it has been suggested that the application of TCSs should start with the lowest potency agents and should take the duration of usage into consideration. Application is recommended once or twice daily within a period of several weeks, depending on the different potencies of the agents, and prolonged use is not recommended [76].

B. Topical calcineurin inhibitors (TCIs), such as pimecrolimus and tacrolimus function by binding to the intracellular protein macrophilin-12, suppressing calcineurin activity, and inhibiting mast cell and neutrophil activation [77]. Pimecrolimus is indicated for use in mild to moderate AD, while tacrolimus is indicated for use in moderate to severe AD. Compared with pimecrolimus, tacrolimus has superior efficacy due to its greater affinity for FK506-binding proteins and better long-term results when used as monotherapy. A meta-analysis, including 14 trials involving 7376 children and adults with AD, demonstrated that both TCIs have favorable efficacy but more adverse events than TCIs [78]. Mild to moderate burning sensations and pruritus are two common adverse events and are generally resolved within one week of treatment initiation, for treatment using both tacrolimus and pimecrolimus.

#### 3.2.2. Systemic Therapeutics

A. An oral antihistamine may provide some benefits in certain circumstances; histamine is considered to be one of the main inducers of pruritus in AD [67]. Some studies have suggested that only short-term use of first-generation antihistamines, also known as sedative antihistamines, may be effective in AD because their sedative effect improves sleep quality [79]. A study compared the use of second-generation antihistamines with and without TCSs in AD patients. The results showed a notable improvement in pruritus and a decrease in blood histamine and tryptase levels in both groups. Another randomized control trial also revealed a significant reduction in pruritus when treating AD patients with a combination of TCSs and fexofenadine, a second-generation antihistamine [80]. According to these studies, it seems that both first- and second-generation antihistamines may be helpful for the treatment of AD.

B. Immunosuppressive medications, as second- or third-line treatment of AD, include cyclosporine, methotrexate, azathioprine, and mycophenylate mofetil. The mechanisms of these agents differ, but their final action is to prevent the activation of T and/or B cells. Before using these immunosuppressive agents, the diagnosis should be confirmed, and other skin diseases, including allergic contact dermatitis and mycosis fungoides, should be ruled out. It is also important to ensure compliance with previous therapies. The selection of these agents should be individualized, as each has its own clinical efficacy and adverse effects [68].

C. Phototherapy utilizes UV light to treat skin diseases and functions primarily by suppressing the expression of cytokines, including IL5, IL13, and IL31, as well as by reducing the numbers of T cells and DCs [81]. Phototherapy also increases microbial diversity on the skin and decreases the proportion of *S. aureus* in AD patients [82]. It produced clinical improvement in, and was well tolerated by, both psoriasis and AD patients [83]. Current types of phototherapy for AD include broadband UVB, narrowband UVB, and UVA-1 therapy, as well as UVA therapy plus 8-methoxypsoralens (PUVA), 308 nm excimer laser (EL), and full-spectrum light (FSL). However, only a few long-term clinical trials have been performed for these modalities. In a systemic review that investigated the efficacy of different modalities of phototherapy in AD, narrowband UVB and UVA-1 were two effective phototherapies for moderate to severe AD [84]. Phototherapy is a valid second-line treatment for both psoriasis and AD patients who have not responded to first-line topical treatment. As no one modality is superior to all, and with a risk of photocarcinogenesis induced by newer modalities, more studies investigating the long-term effects of phototherapy are warranted [85].

#### 3.2.3. Emerging Therapies

A. Dupilumab is a fully human monoclonal IgG4 antibody that binds to the α subunit of the IL4 receptor, subsequently blocking the downstream signaling of IL4 and IL13. The effects of dupilumab include downregulation of inflammatory mediators and markers of proliferation, as well as upregulation of structural lipid metabolism and epidermal barrier proteins that contribute to the normalization of skin [69]. Two phase III randomized control trials revealed that dupilumab had extremely high effectiveness, as well as a favorable safety profile when compared to a placebo in patients with moderate to severe AD [86]. In a one-year randomized, double-blinded, placebo-controlled, phase III trial, patients with moderate to severe AD were randomly assigned to receive 300 mg subcutaneous dupilumab (once weekly or once every two weeks in concert with TCSs) or a placebo in concert with TCSs. The results showed that dupilumab added to standard TCSs is a safe and effective treatment for moderate to severe AD [87].

B. Tralokinumab and lebrikizumab are two IL13-targeting antibodies. Tralokinumab showed great therapeutic efficacy, and had an acceptable safety profile, in treating moderate to severe AD in a phase IIb trial [70]. Lebrikizumab also decreased disease severity and symptoms in moderate to severe AD patients, when combined with TCSs [88].

C. IL31 plays a crucial role in AD. It has been found that overexpression of IL31, independent of the expression of mast cells and lymphocytes, induces pruritus and clinical features in AD patients [89]. Nemolizumab, an inhibitor of IL31, seems to be a promising alternative novel treatment that significantly reduces pruritus and alleviates the symptoms and signs of AD. In two phase II randomized control trials, nemolizumab was well tolerated and efficacious in patients with moderate to severe AD [71]. It also resulted in notable improvements in work productivity for, and reduced activity impairment in, patients with AD [90].

D. Fezakinumab is an IL22 antagonist. IL22, produced mainly by Th22 cells, is the key driver of AD and plays an important role in the expression of pro-inflammatory cytokines while inhibiting KC differentiation [91]. It showed progressing clinical improvements, with acceptable safety profiles in treating moderate to severe AD patients. However, treatment with fezakinumab was most significant for severe AD patients [72]. As such, fezakinumab may be a reasonable treatment choice for patients with severe AD.

E. Secukinumab, a monoclonal antibody directly targeting IL17A, is an approved treatment for psoriasis. IL17A has also been found to increase in peripheral blood and in the lesions of patients with AD. The role of secukinumab in the treatment of AD is currently under evaluation in a phase II study [73].

F. Crisaborole is a topical inhibitor of phosphodiesterase 4 (PDE4), which increases intracellular cyclic adenosine monophosphate. Subsequently, it also suppresses the production of inflammatory cytokines and chemokines, including IFNγ, TNFα, IL2, IL5, and IL10. It also has direct effects on KCs, improving terminal differentiation and preventing hyperplasia in AD patients [92]. Two phase III studies enrolled patients at or above the age of two years, with mild to moderate AD. Patients were randomly assigned to two groups: those who received an application of crisaborole twice daily for 28 days, and those who received a placebo vehicle. Significant differences were noted between the efficacies of crisaborole and the vehicle, including different amounts of reduction of AD signs and symptoms and different amounts of improvement in pruritus and disease severity (AD-301 and AD-302). Application site pain was the only adverse effect reported, with 76.7% of such reports occurring on the first day of application. A total of 77.6% of these complaints were resolved within 1 day of onset [93]. An extension of this study, which included patients from the previous two studies (AD-301 and AD-302), demonstrated that crisaborole had few treatment-related adverse effects over a period of 48 weeks [94]. Due to its favorable safety profile and its ability to improve cases of mild to moderate AD, crisaborole seems to be a promising treatment for AD.

G. Recent evidence has demonstrated that activation of Janus kinase (JAK)-STAT signaling involves the mediation of downstream inflammatory cytokines within the lesional skin of AD patients. JAK inhibitors have immunosuppressive and anti-proliferative effects, as they inhibit JAK-STAT signaling [95]. Baricitinib, an oral selective inhibitor of JAK1 and JAK2, improved pruritus and sleep loss when combined with TCSs in cases of moderate to severe AD, but its efficacy as a monotherapy is lacking. Tofacitinib ointment is a topical form of JAK inhibitors. A phase II trial showed that tofacitinib had great efficacy, early onset of effects, and an acceptable safety profile when compared with the placebo vehicle [74]. JAK inhibitors seem to be effective alternatives to traditional treatment, and more studies on long-term efficacy and safety are warranted (Table 1).

## 4. Conclusions

**Figure 1:**
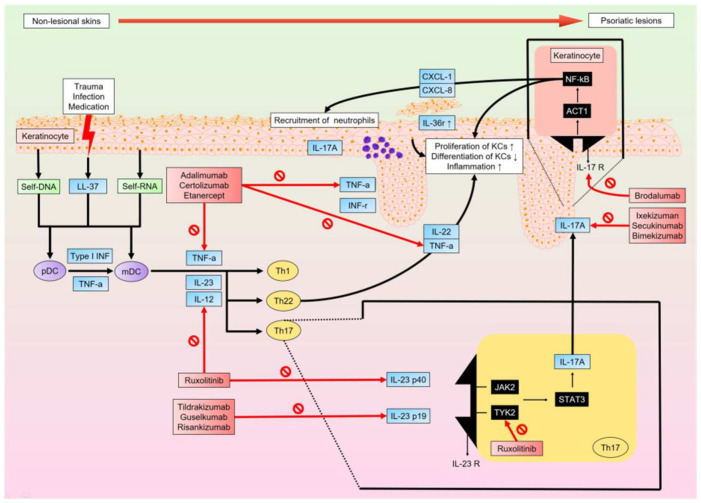
When non-lesional skin is damaged by trauma, infection, and/or medications, KCs release antimicrobial peptides, LL-37, and nucleic acids (self-DNA and self-RNA). LL-37-self-DNA and LL-37-self-RNA complexes stimulate pDCs and mDCs, respectively. pDCs also stimulate mDCs via Type I IFN and TNFα. mDCs produce TNFα, IL12, and IL23 to recruit and activate Th1, Th22, and Th17 cells. They further produce cytokines that upregulate KC proliferation and downregulate KC differentiation, thereby causing inflammation. Regulated primarily by IL23, Th17 cells produce IL17A, which acts on KCs to increase the production of chemokines (CXCL-1 and CXCL-8), which in turn recruit neutrophils and induce transcription of multiple proinflammatory genes. Lesional skin will sustain the inflammatory condition through the cycle associated with the TNFα-IL23-IL17 axis. A variety of novel biologics targeting cytokines of this axis are effective in blocking the inflammatory cycle and improving the clinical condition. pDC, plasmacytoid dendritic cell; mDC, myeloid dendritic cell; IFN, interferon; TNFα, tumor necrosis factor-a; IL, interleukin; JAK, Janus kinase; TYK2, tyrosine kinase 2; STAT, signal transducer and activator of transcription; NF-κB, nuclear factor kappa-light-chain-enhancer of activated B cells.

The development of target therapies for psoriasis depends on our understanding of its complex, multifactorial pathogenesis. Existing evidence strongly supports the thought that the TNFα-IL23-IL17 axis is the main pathway resulting in immune dysregulation. Moreover, biologics that target these three cytokines of the main pathway have been found to sufficiently block the cycle of inflammatory conditions. Although there are still some missing factors in our understanding of the complete pathophysiology of the condition (for example, the role of the microbiome and the complex genetic expression), newly developed biologics show promising outcomes for patients with psoriasis (Figure 1).

**Figure 2:**
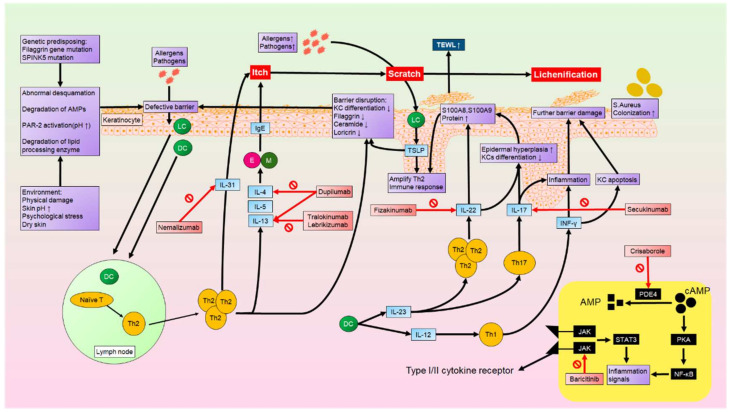
Genetic predisposition and environmental factors can result in skin defects, such as abnormal desquamation and degradation of antimicrobial peptides. Allergens and pathogens can then penetrate through the defective skin barrier, upon which they are recognized by LCs and DCs. LCs and DCs induce the activation and accumulation of Th2 cells from lymph nodes. Th2 cells produce cytokines such as IL4, IL5, IL13, and IL31. These cytokines activate Ms and Es, which further produce IgE. IgE and IL31 then contribute to itching sensations. Scratching the skin causes lichenification and injury, which increases transepidermal water loss and allows more allergens and pathogens to penetrate the skin barrier.

On the other hand, our understanding of AD has progressed through the identification of critical immune cells and cytokines, thereby improving our understanding of multiple different phenotypes that affect different populations. Apart from the previously known Th1 and Th2 immune responses, numerous vital immune cells and cytokines have now been found in AD pathogenesis, such as Th17, Th22, IL31, and TSLP. These findings enable the development of novel biologics targeting the key factors driving the disease. With efficient clinical improvement and fewer systemic side effects, many biologics are preferable to systemic treatment, and different populations can choose different biologics that specify the key mediator in their particular AD phenotypes (Figure 2).

## Figures and Tables

**Table 1:** The internal medicine treatment and target for psoriasis and AD.

Disease and Treatment	Target or Gene	Reference
**Psoriasis**		
**Interleukin 23-targeted therapies**		
Tildrakizumab	IL23 p19	[51]
Guselkumab	IL23 p19	[52]
Ustekinumab	p40	[54]
Risankizumab	IL23 p19	[55]
**Interleukin 17-targeted therapies**		
Brodalumab	IL17	[56]
Xekizumab	IL17A	[57]
Secukinumab	IL17A	[58]
Bimekizumab	IL17A, IL17F	[59]
**Tumor necrosis factor-targeted therapies**		
Adalimumab	p55, p75	[61]
Certolizumab	TNF-α	[62]
Etanercept	TNF-α	[64]
**Atopic dermatitis**		
**Anti-histamine**		[67]
**Immunosuppressive drug**		
Cyclosporine		
Methotrexate		
Azathioprine		
Mcophenylate mofetil		[68]
**Emerging therapies**		
Dupilumab	IL4, IL13.	[69]
Tralokinumab, Lebrikizumab	IL13	[70]
Nemolizumab	IL31	[71]
Fezakinumab	IL22	[72]
Secukinumab	IL17A	[73]
Baricitinib	JAK1, JAK2	[74]
Upadacitinib	JAK	[74]

AD, atopic dermatitis; IL, interleukin; TNF, tumor necrosis factor; JAK, Janus kinase.

## Data Availability

Not applicable.

## Abbreviations

ACT1	actin-1
ACH	acrodermatitis continua of Hallopeau
AD	atopic dermatitis
Alpha-NAC	alpha chain of the nascent polypeptide-associated complex
AMPs	antimicrobial peptides
cAMP	cyclic adenosine monophosphate
CARD	caspase recruitment domain
CCL17/TARC	C-C motif chemokine ligand 17/thymus- and activation-regulated chemokine
CXCL	chemokine (C-X-C motif) ligand
E	eosinphil
EL	excimer laser
FLG	fliaggrin
FKBP	FK506 binding protein
FSL	full-spectrum light
GM-CSF	granulocyte/monocyte colony-stimulating factor
HIV	human immunodeficiency virus
HLA-Cw6	human leukocyte antigen-Cw6
IFN	interferon
Ig	immunoglobulin
IL	interleukin
ISAAC	International Study of Asthma and Allergies in Childhood
JAK2/TYK2	Janus kinase 2/Tyrosine kinase 2
KCs	keratinocytes
KLK	Kallikrein-like peptidase
LC	Langerhan cell
lncRNA	long noncoding RNA
M	mast cell
mDC	myeloid dendritic cell
MHC	major histocompatibility complex
miRNA	microRNA
NB UVA/UVB	narrow band UVA/UVB
NF-κB	nuclear factor-kappa-light-chain-enhancer of activated B cells
PASI	psoriasis area severity index
PAR2	protease-activated receptor 2
pDC	plasmacytoid dendritic cell
PDE4	Phosphodiesterase-4
PPP	palmoplantar pustulosis
PRINS	psoriasis associated RNA induced by stress
PSORS1	psoriasis susceptibility gene 1
PUVA	psoralen and ultraviolet A
PWAR6	Prader Willi/Angelman region RNA 6
RCT	randomized controlled trial
S. aureus	Staphylococcus aureus
SCORAD	scoring atopic dermatitis
SPINK5	Serine protease inhibitor Kazal type 5
STAT	signal transducers and activators of transcription
S100A8/A9	S100 calcium-binding protein A8/A9
TCIs	topical calcineurin inhibitors
TCSs	topical corticosteroids
TEWL	trans epidermal water loss
Th	T helper
TNF-α	tumor necrosis factor-α
TSLP	thymic stromal lymphopoietin
UVR	ultraviolet radiation
